# Tardigrades in Space Research - Past and Future

**DOI:** 10.1007/s11084-016-9522-1

**Published:** 2016-10-20

**Authors:** Erdmann Weronika, Kaczmarek Łukasz

**Affiliations:** Faculty of Biology, Department of Animal Taxonomy and Ecology, Adam Mickiewicz University in Poznań, Umultowska 89, 61-614 Poznań, Poland

**Keywords:** Extreme conditions, Extremophiles, Space research, Space biology, Tardigrada, Water bears

## Abstract

To survive exposure to space conditions, organisms should have certain characteristics including a high tolerance for freezing, radiation and desiccation. The organisms with the best chance for survival under such conditions are extremophiles, like some species of Bacteria and Archea, Rotifera, several species of Nematoda, some of the arthropods and Tardigrada (water bears). There is no denying that tardigrades are one of the toughest animals on our planet and are the most unique in the extremophiles group. Tardigrada are very small animals (50 to 2,100 μm in length), and they inhabit great number of Earth environments. Ever since it was proven that tardigrades have high resistance to the different kinds of stress factors associated with cosmic journeys, combined with their relatively complex structure and their relative ease of observation, they have become a perfect model organism for space research. This taxon is now the focus of astrobiologists from around the world. Therefore, this paper presents a short review of the space research performed on tardigrades as well as some considerations for further studies.

## What are Tardigrades?

Water bears (Tardigrada), discovered in 1773, are a phylum of small invertebrates belonging to the supertype Articulata. They can be found all over the Earth and can inhabit very diverse environments (from the deepest oceans to mountain tops). Water bears are small, cylindrical invertebrates, up to 2.1 mm in length, and are divided into five segments. The first segment contains the head and the next four each have one pair of unsegmented legs ending (most often) in claws. The tardigrade body is covered with a flexible cuticle, which is smooth or covered with gibbosities, spines or plates. Despite being so small, they have a very complicated internal structure. Water bears have a complete digestive system adapted, depending on the species, to consume algae, bacteria, fungal cells or small invertebrates such as rotifers, nematodes and other tardigrades. They have a well developed nervous system consisting of rings around mouth (brain) and an abdominal chain with segmental ganglia. They also have various sensory organs like papilla, chemoreceptors and eyes. Water bears can be dioecious and bisexual; in the second case, sexual dimorphism is sometimes present (especially in Heterotardigrada). In many species, the phenomenon of parthenogenesis is also present. Fertilisation can be external or internal. The eggs are covered with an additional shell, the smooth or ornamented chorion, and are laid freely (directly into the environment) or in old exuviae. After hatching, tardigrades moult between two to seven times. The young resemble the adults, and they reach sexual maturity after the second or third moult, which takes several days. Adult tardigrades live approximately two to several months (Nelson et al. 2015). Currently, approximately 1,200 tardigrade species are known (Degma et al. 2009–2016; Vicente and Bertolani 2013), although it is estimated that the real number of species is much higher. At present, this phylum is divided into two classes, Eutardigrada and Heterotardigrada. Eutardigrada consists mainly of freshwater and terrestrial species (marine species are extremely rare). The body of eutardigrades is translucent or milky white (but sometimes has different colouration) and is covered with a flexible cuticle and devoid of plates. Among Heterotardigrada, both marine and terrestrial species are present. Their body is covered with a cuticle that produces various kinds of plates (Nelson 2002; Nelson et al. 2015).

## What Makes Them so Special?

As mentioned above, tardigrades live not only in freshwater and marine environments but also in terrestrial habitats. Terrestrial species can be found in mosses, lichens and soil where they are threatened by drying. In this situation, terrestrial species need a thin water film around their bodies in order to stay active. These species have also developed a special skill that protects them against the effects of dehydration: the ability to enter into a cryptobiotic state. There are several types of cryptobiosis: **a)** anoxybiosis, a reaction to lack of sufficient oxygen, **b)** cryobiosis, a reaction to freezing temperatures, **c)** osmobiosis, a reaction to excessive salinity, and the best known type of cryptobiosis, **d)** anhydrobiosis, a reaction to a lack of liquid water in the environment (Kinchin 2008). In the anhydrobiotic state, the metabolic activity of tardigrades drops to a very low level (Pigoń and Węglarska 1955). This latent state can occur at the egg stage as well as in adults and can be repeated multiple times (Kinchin 2008). Anhydrobiosis gives tardigrades resistance to a lack of water, but also to a number of physical factors such as high temperature, radiation or different kinds of chemicals, such as ethanol, hydrogen sulphide and carbon dioxide (Kinchin 1994; Ramlov and Westh 2001; Wełnicz et al. 2011; Guidetti et al. 2012). Not all Tardigrada species show equal resistance to drying out, as there are differences in drying tolerance even between populations of the same species (Jönsson et al. 2001; Horikawa and Higashi 2004). The entrance into anhydrobiosis is preceded by a preliminary phase, during which the tardigrade body undergoes a series of metabolic and anatomical changes that are necessary to survive the unfavourable conditions. The changes are easiest to observe as a shrinkage of the body (forming numerous folds which reduce the body’s surface area), namely the adoption of the tun formation (Baumann 1922). The tun form reduces the surface for evaporation and thus slows down the loss of liquid water (transpiration is reduced by about 50 %) (Wright 1989). The tun state also prevents the destruction of the internal and external organs during the drying process (Crowe 1975). When all free water evaporates from the tardigrade body, it begins the process of replacing the water bound to macromolecules. The lost water is replaced with bioprotectants such as trehalose, which protects macromolecules, such as nucleic acids and proteins, from losing their proper structure (Kinchin 2008). If the macromolecule structure is damaged, the cell dies. It is not certain how trehalose contributes to the protection of membrane proteins. It is also possible that the trehalose hydroxyl groups interact with hydrogen atoms replacing the same evaporating water (Kinchin 2008). In summary, trehalose is responsible for stabilising proteins, membrane lipids and nucleic acids (Webb 1964; Crowe 2002). However, it should be also emphasised that some tardigrades synthesise trehalose on a very low level (Wang et al. 2014). The other molecules involved in tardigrade cell protection during anhydrobiosis are LEA (late embryogenesis abundant), HSP (heat shock proteins), CAHS (cytoplasmic abundant heat soluble), SAHS (secretory abundant heat soluble) and aquaporin proteins (Förster et al. 2009; Yamaguchi et al. 2012; Grohme et al. 2013; Guidetti et al. 2011, 2012; Wełnicz et al. 2011). The LEA proteins have similar functions to trehalose, but in addition to protecting the cell membranes and proteins, they can also act as a hydration buffer and sequester ions. They can also be responsible for cell structure protection through renaturation of unfolded proteins (Tunnacliffe and Wise 2007). In turn, the HSPs could work as molecular chaperons (Goyal et al. 2005) and participate in protein folding, and inhibiting protein aggregation. So far, it has been proven that this type of protein (presumably the Hsp70-90 proteins family) are produced and stored in organisms going into anhydrobiosis, but their role during desiccation is still uncertain (Ramlov and Westh 2001; Reuner et al. 2010; Wełnicz et al. 2011). The CAHS and SAHS proteins probably form a molecular shield in water-deficient conditions (Yamaguchi et al. 2012). Aquaporin proteins may play a minor role during anhydrobiosis by fine-tuning water transport and greatly increasing membrane permeability (Grohme et al. 2013). Also, possession of the ROS (Reactive oxygen species) scavenging enzymes could represent a crucial strategy to avoid damages during desiccation in anhydrobiotic tardigrades (Rizzo et al. 2010; Rebecchi 2013). However, most of the bioprotectants and mechanisms that protect tardigrade cells during cryptobiosis are still poorly understood or completely unknown.

### Toughest Animals on Earth

The ability to enter into a state of anhydrobiosis (which distinguishes water bears from most other organisms) lets tardigrades resist many unfavourable environmental factors (Rebecchi et al. 2007). Moreover, tardigrades are able to survive in an inactive form for many years (from nine to 20 years in natural conditions) (Guidetti and Jönsson 2002; Rebecchi et al. 2006; Guidetti et al. 2012). Interestingly, the death of individuals that are in a long anhydrobiotic state is mostly caused by the drying process itself and not by the aging process. This phenomenon can be explained by the so-called “Sleeping Beauty” model, first reported in rotifers (Ricci 2001; Segers and Shiel 2005). At the time when the model was confirmed for rotifers, this kind of shift in the age of anhydrobiotic animal was also supposed for tardigrades (Hengherr et al. 2008). On the other hand, it was also proven that during anhydrobiosis, cell damages accumulate with time (Rebecchi et al. 2009a). It is possible that such damages are accumulated in proportion to the time spent in anhydrobiosis and lead to animal death, even though desiccation itself does not seem to have an effect on tardigrade longevity and ageing (Guidetti et al. 2011). Water bears are also very resistant to extreme temperatures, and they can survive from −272.8 °C (Becquerel 1950) to about 150 °C (up to 15 min) (Rahm 1923, 1924, 1926). Resistance to low temperatures was investigated repeatedly during research on anhydrobiosis and on cryobiosis. One of the first studies showed that many different species of Tardigrada withstand immersion in liquid air (*ca.* -190 °C), liquid nitrogen (*ca.* -253 °C) and liquid helium (*ca.* -272 °C) (Rahm 1923, 1924, 1926). Other studies demonstrated that some species inhabiting the Arctic soil can survive up to six years (74 months) at −80 °C (Newsham et al. 2006). These small invertebrates also exhibit significant resistance to low and high atmospheric pressures (from 200 to 280 hPa to 7,500 MPa) (Jönsson et al. 2008; Ono et al. 2008). Tardigrades in an anhydrobiotic state are also resistant to high doses of ionising radiation and X-rays (*ca.* 5000 GY) (May et al. 1964; Horikawa et al. 2006). Some individuals are even able to survive very high doses of ultraviolet radiation (between 75 and 88 kJ m^2^) (Altiero et al. 2011). Water bears are resistant to physical stressors as well as some chemical stressors such as hydrogen sulphide, carbon dioxide, ethanol (for *ca.* 10 min) and 1-hexanol (Baumann 1922; Ramlov and Westh 2001).

Unlike the other multicellular extremophiles, water bears are not only resistant in the anhydrobiotic state but also in the active state. Active tardigrades are able to survive in temperatures of about 38 °C (Li and Wang 2005; Rebecchi et al. 2009b) and −196 °C (Ramlov and Westh 2001). They also exhibit a significant resistance to high atmospheric pressures (up to 100 MPa) (Seki and Toyoshima 1998). It is also known that tardigrades in the active state are almost as resistant to radiation as in anhydrobiosis (Jönsson et al. 2005; Horikawa et al. 2006 2009; Altiero et al. 2011).

### Tardigrades in Space Research and Space Missions

All of the above mentioned features of tardigrades caused scientists to consider them in the context of space research. In 1964, in the article “Actions différentielles des rayons x et ultraviolets sur le tardigrade *Macrobiotus areolatus*”, it was suggested for the first time that tardigrades, due to their enormous resistance to radiation, could be model animals for space research (May et al. 1964). Thirty-seven years later, in the article “Tardigrades as a potential model organism in space research”, Bertolani et al. (2001) suggested a similar concept. At the same time, studies focused on the phenomenon of cryptobiosis in tardigrades were conducted, revealing still greater resistance of this amazing animal to many unfavourable factors encountered in outer space. In 2007, Jönsson, based on knowledge resulting from these studies, showed that tardigrades can be suitable model organisms for astrobiological studies because of their ability to dehydrate, extreme temperature tolerance and radiation resistance (Jönsson 2007). Since that time, numerous articles suggesting that tardigrades can be used in space research have been published (Horikawa et al. 2008; Rebecchi et al 2010a). The latest paper from early 2012 indicated that tardigrades are an excellent model for space research (Guidetti et al. 2012). While that paper emphasises the previously known extraordinary resistance of tardigrades, it also suggests that there is a greater complexity to tardigrade organisms. The authors repeatedly emphasise that the complex structure of tardigrades allows extrapolation of the results of such studies to vertebrates (including humans). In conjunction with their small body size, their relative ease to culture and obtain offspring further enhances their importance as a potential model species (Guidetti et al. 2012). In 2008, Horikawa et al. proposed *Ramazzottius varieornatus* Bertolani & Kinchin, 1993 as a model species in astrobiology research. In their paper they described a methodology for breeding this species under laboratory conditions. They also described the life history of this species and identified characteristics required to consider this particular species of tardigrade as a model (Horikawa et al. 2008). In the same year, it was also suggested that tardigrades could travel through space in a large meteorite and could probably confirm the theory of panspermia (Ono et al. 2008).

Based on researcher suggestions, a few space programmes focused on tardigrades were started and finished in recent years. In 2007, three projects were conducted during the FOTON-M3 mission studies. The Tardigrade Resistance to Space Effects (TARSE) Project was the first one involved in the mission of FOTON-M3. Its aim was to analyse the impact of environmental stress, life history traits and DNA damages in space (on board the spacecraft) on eutardigrade *Paramacrobiotus richtersi* (Murray, 1911). In this project active and anhydrobiotic tardigrades were exposed to radiation in microgravity conditions. Both active and inactive individuals had high survival rates with no induction of HSPs while showing an induction of the antioxidant response (Rebecchi et al. 2009c, 2010b, 2011a). The next project involved in the mission of FOTON-M3 was TARDIS (Tardigrada In Space). The main goal of this project was to check whether tardigrades from two species, *Milnesium tardigradum* Doyère, 1840 and *Richtersius coronifer* (Richters, 1903), were able to survive conditions of open space. The experiments showed that tardigrades can survive exposure to the space vacuum, but the addition of factors such as ultraviolet solar radiation, ionising solar radiation and galactic cosmic radiation significantly reduced their survival rate (Jönsson, et al. 2008). In the third project from the FOTON-M3 mission, RoTaRad (Rotifers, Tardigrades and Radiation), scientists examined effects on initial survival, long-term survival and fecundity of selected species of limno-terrestrial tardigrades in extreme stress conditions (mainly cosmic radiation) (Persson et al. 2011). Next was the Endeavour mission in 2011 and the project TARDIKISS (Tardigrades in Space). The main aim of this project was to broaden our knowledge of life history traits and mechanisms of repairing structural DNA damage during exposure to space flight stresses (Rebecchi et al. 2011b; Vukich et al. 2012). The first results showed that microgravity and cosmic radiation did not significantly affect the survival rate of tardigrades (Rebecchi et al. 2011b; Vukich et al. 2012). However, Rizzo et al. (2015) showed a significant difference in activities of ROS scavenging enzymes, the total content of glutathione and the fatty acid composition between tardigrades sent into space and control animals on Earth. The last space research project involving tardigrades was the Phobos Life Project. It was a part of the Phobos Ground Mission. The goal of this project was to study how the living organisms survive during space flight conditions. Scientists wanted to test the viability of selected organisms during an interplanetary flight lasting approximately 34 months and verify the theory of panspermia (http://www.forum.kosmonauta.net/index.php?topic=585.35;wap2). For this mission, the same organisms already used in other space experiments, and well-known to be radiation resistant, were used. They represented all three domains of life (Bacteria, Eukaryotes and Archaea). A total of 10 different taxa were used (species or strains), including three species of tardigrades: *M. tardigradum*, *R. coronifer* and *Echiniscus testudo* (Doyère, 1840). Unfortunately, the experiments were not successful because the spacecraft carrying the whole apparatus crashed and burned over the South Pacific Ocean on January 15th 2012.

### Further Perspectives

As demonstrated above, we already know quite a lot on the limits of endurance of tardigrades regarding various stress factors. However, we still do not know the exact mechanisms of action that protect and repair their bodies in unfavourable conditions. The understanding of these mechanisms and knowledge of the responsible genes is an important step in astrobiological studies, especially if it can be extrapolated to vertebrates (including humans).

There are many possible future directions of astrobiological research regarding tardigrades. For example, one experiment could evaluate the ability of tardigrades to survive in a simulated atmosphere of certain celestial bodies in our solar system (such experiments simulating Martian conditions were conducted on bacteria, cyanobacteria, lichens and also tardigrades (Cockell 2005; Johnson et al. 2011; Rebecchi et al 2010b; Smith et al. 2009; Vera et al. 2010; 2014). This is interesting because a few of the celestial bodies in our solar system may periodically exhibit micro-environmental conditions appropriate for the survival of certain extremophiles. For example, it is well known that Martian soil contains water (Mitrofanov et al. 2014), and in some regions of Mars, in the summer periods, temperatures up to 20 °C were recorded (NASA, official webpage). Even if all environmental conditions are not entirely favourable for life, tardigrades are quite resistant in both the anhydrobiotic and the active state. Moreover, organisms which provide nourishment for tardigrades (e.g. bacteria, algae, rotifers or nematodes) are just as resistant as water bears (Guidetti and Jönsson 2002; Rettberg et al. 2002; Islam and Schulze-Makuch 2007; Meeßen et al. 2013). However, these relatively beneficial life periods are interrupted by periods of very unfavourable conditions for living organisms. This is where cryptobiosis has a potential and very important role. The ability to enter into cryptobiosis is helpful not only for travelling for long cosmic distances, but also for providing a possibility of surviving long periods when environmental conditions are unfavourable. This could enable researchers to determine whether tardigrades can survive and live on other planets in the solar system or on their moons. We should also continue studies on tardigrade resistance to combined stress conditions such as the combined effects of cosmic radiation and microgravity, or low temperature and the presence of harmful chemicals. Such studies would help determine the limits of survival of Earth’s multicellular organisms. This is very interesting especially in the context of searching for life on other planets and moons.
